# Structural enzymology comparisons of multifunctional enzyme, type‐1 (MFE1): the flexibility of its dehydrogenase part

**DOI:** 10.1002/2211-5463.12337

**Published:** 2017-11-06

**Authors:** Prasad Kasaragod, Getnet B. Midekessa, Shruthi Sridhar, Werner Schmitz, Tiila‐Riikka Kiema, Jukka K. Hiltunen, Rik K. Wierenga

**Affiliations:** ^1^ Biocenter Oulu and Faculty of Biochemistry and Molecular Medicine University of Oulu Finland; ^2^ Theodor Boveri Institute of Biosciences (Biocenter) University of Würzburg Germany

**Keywords:** CoA, crotonase, dehydrogenase, NAD, substrate channeling

## Abstract

Multifunctional enzyme, type‐1 (MFE1) is a monomeric enzyme with a 2E‐enoyl‐CoA hydratase and a 3S‐hydroxyacyl‐CoA dehydrogenase (HAD) active site. Enzyme kinetic data of rat peroxisomal MFE1 show that the catalytic efficiencies for converting the short‐chain substrate 2E‐butenoyl‐CoA into acetoacetyl‐CoA are much lower when compared with those of the homologous monofunctional enzymes. The mode of binding of acetoacetyl‐CoA (to the hydratase active site) and the very similar mode of binding of NAD ^+^ and NADH (to the HAD part) are described and compared with those of their monofunctional counterparts. Structural comparisons suggest that the conformational flexibility of the HAD and hydratase parts of MFE1 are correlated. The possible importance of the conformational flexibility of MFE1 for its biocatalytic properties is discussed.

**Database:**

Structural data are available in PDB database under the accession number 5MGB.

**Abbreviations**
AcAc‐CoA: acetoacetyl‐CoA
HAD: 3S‐hydroxyacyl‐CoA dehydrogenase
HsHAD: the human, mitochondrial, dimeric, monofunctional HAD
MES: 2‐(N‐morpholino) ethanesulfonic acid
MFE1: multifunctional enzyme, type‐1
PIPES: piperazine‐N,N′‐bis(2‐ethanesulfonic acid)
rpMFE1: the rat, peroxisomal, monomeric MFE1
Tris: tris(hydroxymethyl)‐aminomethane

In higher eukaryotes, the fatty acid degradation by β‐oxidation is located in peroxisomes 1, 2, 3 as well as in mitochondria 4, 5, 6. The peroxisomal β‐oxidation pathway catalyzes the degradation of the very long straight chain, 2‐methyl branched chain, as well as bile acid intermediates and polyunsaturated fatty acid chains, whereas the mitochondrial β‐oxidation degrades predominantly the saturated, long‐, medium‐, and short‐chain fatty acids. Peroxisomal β‐oxidation is catalyzed by monofunctional and multifunctional enzymes. The first step of the peroxisomal β‐oxidation pathway (Fig. 1), the acyl‐CoA oxidase reaction, and the fourth (last) step, the thiolase reaction, are catalyzed by monofunctional enzymes. The peroxisomal multifunctional enzyme, type‐1 (MFE1) catalyzes the second and third steps. MFE1 is a monomeric enzyme 7. The N‐terminal region catalyzes the second step of β‐oxidation, which is the hydratase reaction (EC 4.2.1.17), as well as the Δ^3^,Δ^2^‐enoyl‐CoA isomerase reaction (EC 5.3.3.8) 8, 9. The third step of the β‐oxidation pathway, the NAD‐dependent dehydrogenation reaction of 3‐hydroxyacyl‐CoA (EC 1.1.1.35), is catalyzed by the C‐terminal part of MFE1. MFE1 is also known as the L‐bifunctional protein, because of the chirality of the 3‐hydroxy intermediate, being 3S‐hydroxyacyl‐CoA 10. MFE1 has been proposed to play a key role in the metabolism of dicarboxylic fatty acids 11, 12. MFE1 is one of the most abundant enzymes in mammalian peroxisomes; nevertheless, MFE1 deficiency diseases have not yet been described, suggesting that MFE1 is important under as of yet uncharacterized metabolic stress.

**Figure 1:**
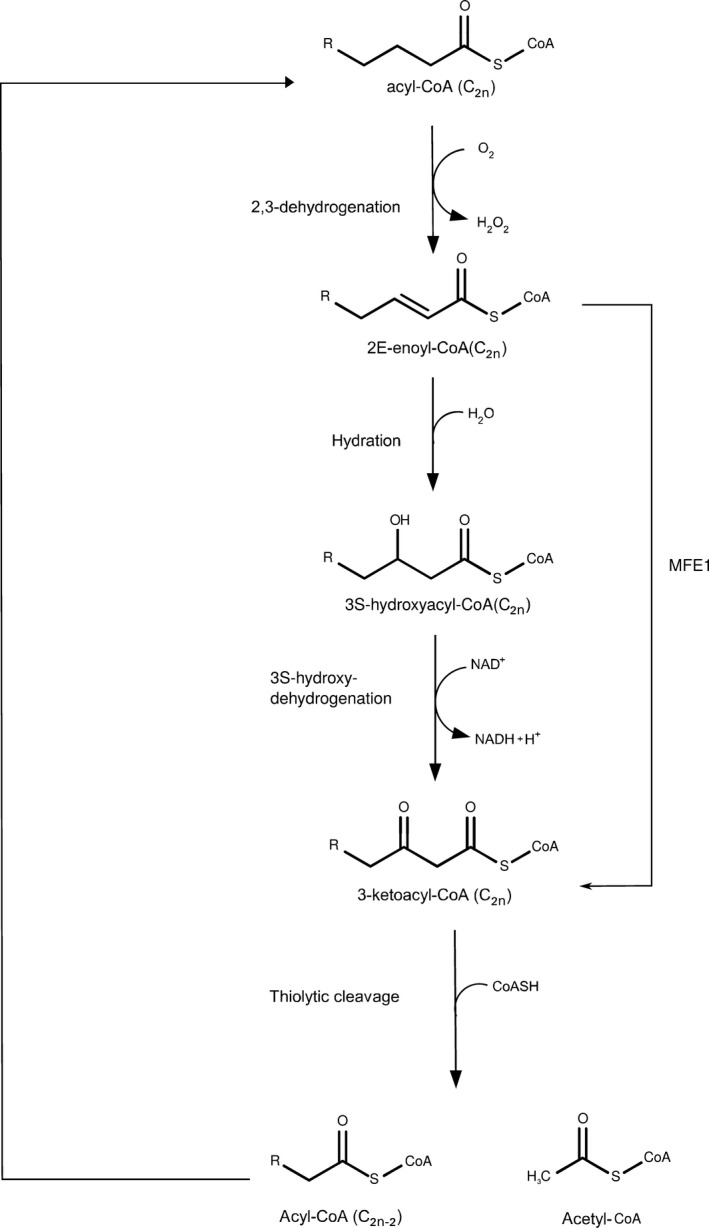
The four reactions of the peroxisomal β‐oxidation pathway. MFE1 catalyzes the second (hydration) and the third (3S‐hydroxyacyl‐CoA dehydrogenation) reaction.

Substrate specificity studies have shown that MFE1 can catalyze the hydration of long‐chain linear acyl chains as well as of the bulky bile acid intermediates. The latter acyl moieties also have a 2‐methyl group. Interestingly, the chiral specificity for the 2‐methyl group is different for the hydratase active site and the dehydrogenase active site 13, 14, 15. Schulz and coworkers have shown that for the substrate 2E,4E‐decadienoyl‐CoA efficient channeling of the intermediate between the two active sites is observed 16. Substrate channeling is an intriguing property of some enzyme systems 17, being well established for the mitochondrial β‐oxidation pathway 18. However, at the molecular level, the substrate channeling mechanism of such bulky and polar, substrate molecules is poorly understood 19.

The crystal structure of rat peroxisomal MFE1 (rpMFE1, 722 residues) (complexed with CoA) has been reported 20. Five domains (A, B, C, D, and E) have been recognized in this fold (Fig. 2, Fig. S1). Its N‐terminal part (catalyzing the hydratase reaction) has the crotonase fold (formed by the A and B domains), like the well‐studied monofunctional Δ^2^‐enoyl‐CoA hydratases 21. The C‐terminal part (catalyzing the dehydrogenase reaction) adopts the 3S‐hydroxyacyl‐CoA dehydrogenase (HAD) fold, like that described for the monofunctional human HAD (HsHAD) 22. This HAD part has two lobes, a NAD‐binding domain (the C domain) and a substrate binding region (the D/E domains) (Fig. 2). The D and E domains interact tightly with each other and have a very similar fold. The N‐terminal and C‐terminal parts of MFE1 are connected via a helical linker region. This linker region (domain B) functionally belongs to the crotonase fold, being the helix‐10 of this fold, but structurally it belongs to the C‐terminal HAD part (Fig. 2). The E domain has stabilizing interactions with this linker helix via hydrophobic interactions (by residues of EH2) and via a salt bridge (between Arg657 of the support loop (domain E) and Glu274 of the linker helix) 20. The two active sites are about 40 Å apart, separated by a solvent‐exposed tunnel. This tunnel is lined by an excess of positively charged residues 20, suggesting that substrate channeling of the negatively charged acyl‐CoA molecules via electrostatic interactions with the positively charged tunnel surface could be important, as described for the thymidylate synthase–dihydrofolate reductase bifunctional enzyme 23, 24.

**Figure 2:**
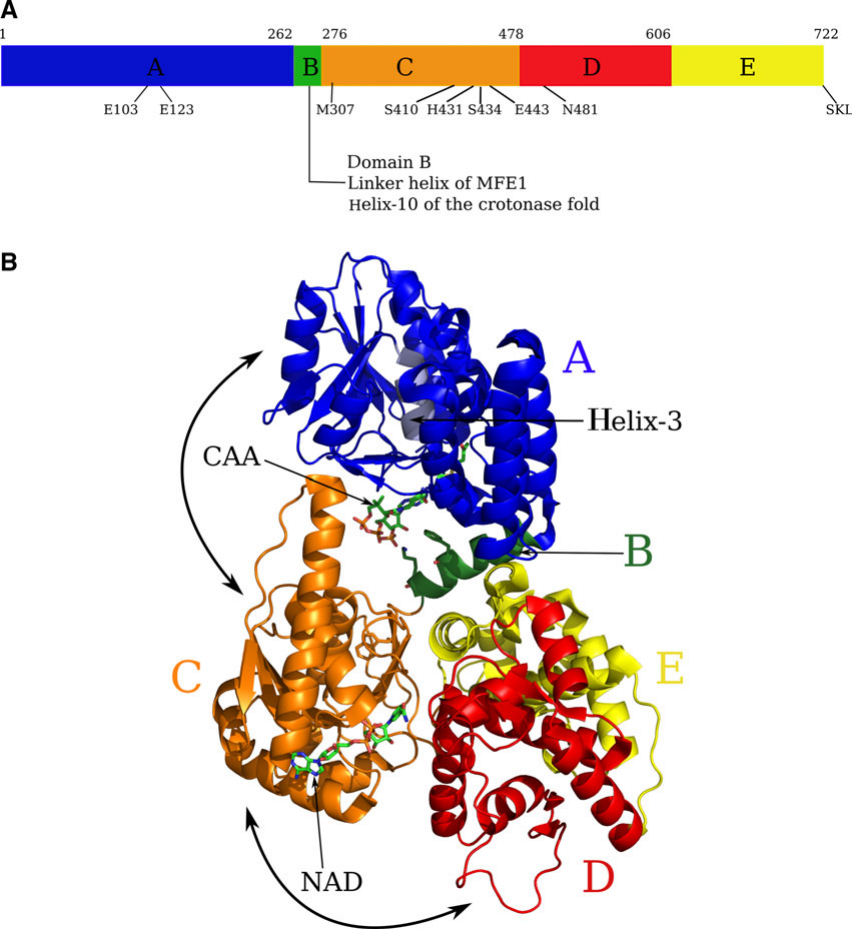
The five MFE1 domains. (A) Schematic division of the MFE1 sequence in five domains. Domain B is also referred to as the linker helix or the helix‐10. Glu103 and Glu123 are the catalytic residues of the A domain. Met307 contacts the nicotinamide moiety of NAD ^+^, and Ser410, His431, Ser434, Glu443, and Asn481 are important catalytic residues of the dehydrogenase domain. (B) The MFE1 fold, color‐coded according to the domains (blue, domain A; green, domain B; orange, domain C; red, domain D; and yellow, domain E). Domains D and E have the same fold and are tightly interacting with each other. The ligands of this structure (5MGB) identify the hydratase active site (AcAc‐CoA, labeled as CAA) and the dehydrogenase active site (NAD ^+^, labeled as NAD ^+^). The active site helix (the labeled ‘helix‐3’ arrow points at its N terminus) of the hydratase domain, between Gly101 and Cys108, is colored light blue. The side chains of Phe271, Lys275, and Trp280 are shown as sticks. Phe271 and Lys275 of the linker helix (domain B) point toward the AcAc‐CoA bound in the hydratase active site. Trp280 is at the C‐terminal end of the linker helix. The bold arrows visualize the two hinge motions, being (a) of domain A with respect to the BCDE domains and (b) of domain C with respect to the D/E domains.

The rpMFE1 Michaelis–Menten kinetic measurements reported here for short‐chain substrates show that the catalytic efficiency of the two active sites of MFE1 is much lower than the efficiency of the corresponding monofunctional enzymes. The structural studies concern the analysis of the structure of rpMFE1 complexed with AcAc‐CoA and NAD^+^. In the available MFE1 crystal structures, there are two molecules in the asymmetric unit. In these two molecules, referred to as molecule A and molecule B, the domains adopt different conformations with respect to each other (Fig. 2). The comparison of these crystal structures suggests that this conformational flexibility of MFE1 is of key importance for the proper functioning of this enzyme.

## Materials and methods

### Expression and purification of rat peroxisomal MFE1

The cloning of the rpMFE1 (UniProt P07896) into pET15b vector has been described previously 20. The expression and purification protocols of rpMFE1 were the same as reported previously 25. At the end of the purification protocol, the concentrated protein was loaded on a Superdex‐200 size exclusion chromatography column which was pre‐equilibrated with gel filtration buffer (10 mm piperazine‐N,N′‐bis(2‐ethanesulfonic acid (PIPES), 50 mm NaCl, pH 6.5). The peak fractions of the protein were pooled, concentrated, and stored at −70 °C.

### Enzyme assays

The enzyme kinetic measurements were taken by following the reaction with the JASCO V660 spectrophotometer (JASCO Corporation, Tokyo, Japan) at 25 °C for 3 min in a 1‐cm cuvette using 0.5 mL assay volumes. In each assay the reaction was initiated by adding the enzyme. The *k*_cat_ and *K*_m_ values were determined using the JASCO software spectramanager. The substrates 2E‐butenoyl‐CoA (for the forward hydratase and dehydrogenase reactions), AcAc‐CoA (for the reverse dehydrogenase reaction), NAD^+^, and NADH were purchased from Sigma (Sigma Aldrich, St Louis, MS, USA).

The 2E‐enoyl‐CoA hydratase activity was measured by monitoring the disappearance of the C‐C double bond of 2E‐butenoyl‐CoA to form 3S‐hydroxybutanoyl‐CoA. The assay buffer contained 50 mm tris(hydroxymethyl)‐aminomethane (Tris) (pH 9.0), 50 mm KCl, and 50 μg·mL^−1^ BSA. The progress of the reaction was monitored at 263 nm, with 95 ng·mL^−1^ present in the cuvette. For calculating the initial rates, an absorption coefficient of 6700 m ^−1^·cm^−1^ was used.

The forward dehydrogenase assay of rpMFE1 uses as substrate 2E‐butenoyl‐CoA, which is converted by the hydratase active site into 3S‐hydroxybutanoyl‐CoA. This then is the substrate of the dehydrogenase active site, which catalyzes the dehydrogenation of 3S‐hydroxybutanoyl‐CoA into acetoacetyl‐CoA in the presence of NAD^+^, being converted into NADH. The reaction mixture contained 50 mm Tris (pH 9), 50 mm KCl, 50 μg·mL^−1^ BSA, and 1 mm NAD^+^. The reaction was carried out with 950 ng·mL^−1^ in the cuvette and the reaction was monitored at 340 nm (ϵ_340 _= 6220 m ^−1^·cm^−1^).

The reverse dehydrogenase assay measures the conversion of the substrate AcAc‐CoA into 3S‐hydroxybutanoyl‐CoA by the dehydrogenase active site of rpMFE1, using as assay buffer 50 mm Tris/HCl, 50 mm KCl, 50 μg·mL^−1^ BSA pH 7.0, and 0.1 mm NADH. The activity was monitored at 340 nm by following the disappearance of NADH with 950 ng·mL^−1^ present in the cuvette.

### Crystallization, data collection, data processing, and structure refinement of the AcAc‐CoA/NAD^+^ complex

The AcAc‐CoA/NAD^+^ complex structure was obtained from a crystal grown at room temperature by cocrystallization in the presence of AcAc‐CoA and NAD^+^ using otherwise the same crystallization protocol as previously described for the CoA‐complexed crystal form 25. Briefly, these crystals were grown at room temperature using the sitting drop method. The protein solution was 8 mg·mL^−1^ in 10 mm PIPES, pH 6.5 and 50 mm NaCl, 2 mm AcAc‐CoA, and 2 mm NAD^+^, and the well solution was 100 mm 2‐(N‐morpholino) ethanesulfonic acid, pH 6.0, 150 mm ammonium sulfate, and 15% PEG4000 (w/v). Before data collection, the crystal was cryoprotected by a brief soak in well solution complemented with 15% glycerol, 2 mm AcAc‐CoA, and 2 mm NAD^+^. The data set of the AcAc‐CoA/NAD^+^ complex was collected at ESRF in Grenoble, France (Table 1). The data set was processed with iMOSFLM 26 and scaled using SCALA 27. The statistics of the data processing are summarized in Table 1. The space group and cell dimensions are the same as previously found for the CoA‐complexed crystal form 20 and there are two molecules per asymmetric unit. The initial phases were obtained by a rigid body refinement by REFMAC5 28 using as a starting model the rpMFE1 model of the CoA‐complexed structure (2X58), in which the solvent and ligand molecules were removed. The structure was refined further with REFMAC5 at 2.8 Å resolution, using NCS restraints. The active site ligands were modeled in the maps using COOT 29 at the end of the refinement. The structure was validated and improved using MolProbity 30 and COOT. The refinement was stopped once the remaining peaks in the electron density maps, at approximately 5 sigma, did not have structural information. The final refinement statistics are summarized in Table 1.

**Table 1:** Data collection and refinement statistics

Crystal form	The AcAc‐CoA/NAD^+^ complex crystal form, with bound AcAc‐CoA and NAD^+^
PDB entry code	5MGB
Beam line	ID14‐2 (ESRF)
Temperature (K)	100
Wavelength (Å)	0.933
Space group	P2_1_2_1_2_1_
Unit cell parameters (Å)	65.23 125.82 223.90
Number of molecules per asymmetric unit	2
*V*_m_ (Å^3^/Da)	2.8
Resolution range (Å)^a^	35.0–2.8 (2.95–2.8)
Completeness (%)^a^	83.8 (84.2)
<*I*/σ(*I*)>^a^	8.0 (2.4)
*R*_pim_ (%)^a^	6.0 (23.0)
Number of unique reflections^a^	38 432 (5578)
Redundancy^a^	3.8 (3.1)
Wilson B factor (Å^2^)	67
Refinement	
Resolution (Å)	33.6–2.8
*R*_factor_ (%)	20.9
*R*_free_ (%)	26.1
Total number of reflections	36 256
Number of protein atoms	11 092
Number of waters	96
Number of other molecules glycerol/sulfate	2/4
Rmsd, protein bonds (Å)	0.008
Rmsd, protein angles (°)	1.3
Rmsd B factor	
Protein main chain A, B (Å^2^)	1.4, 1.4
Protein side chain A, B (Å^2^)	1.5, 1.5
Average B factor	
Protein molecule, A/B (Å^2^)	49/60
A‐hydratase active site ligand (AcAc‐CoA) (Å^2^)	65
A‐dehydrogenase active site ligand (NAD^+^) (Å^2^)	63
B‐hydratase active site ligand (AcAc‐CoA) (Å^2^)	57
B‐dehydrogenase active site ligand (NAD^+^) (Å^2^)	63
Ramachandran plot^b^	
Favored (%)	97.2
Allowed (%)	2.8
Outliers (%)	0

^a^ The numbers in parentheses refer to the outer shell. ^b^ Calculated using Rampage 45.

### Structure analysis

In the AcAc‐CoA/NAD^+^ complex, structure molecules A and B have been built completely from residues −4 to 720 and from residues −1 to 718, respectively. The most flexible regions, having high B factors in both molecules, are loop‐2 of the hydratase domain (near residue 70), the tip of the CH2 helix of the C domain (near residue 355), the substrate specificity loop of domain D (near residue 545), the β‐meander of the D domain (near residue 587), and the loop between domains D and E (near residue 610). The structure of molecule A has been used for the structure analysis, employing programs of the CCP4 package 31, unless stated otherwise.

The rpMFE1 hydratase active site (liganded with AcAc‐CoA) has been compared with the liganded monofunctional hydratase active site (liganded with AcAc‐CoA (1DUB) 32) as well as with the rpMFE1 active site (complexed with CoA (2X58) 20, with 3S‐hydroxydecanoyl‐CoA (3ZWC) 25 and unliganded (3ZW8) 25) by superimposing the rpMFE1 hydratase domain and the hydratase subunit on each other, using the SSM superpositioning option of COOT 33.

For the characterization of the open/closed conformations of the HAD part of rpMFE1, several crystal structures of HsHAD have been used, in particular the ternary complexed, fully closed structure (1F0Y) 22 and the binary complexed structures with NAD^+^ (3HAD) 34 and NADH (1F17) 22. For these comparisons, two superposition protocols (employing the SSM superpositioning option of COOT) were used, being either (a) superimposing the C domain of rpMFE1 on the NAD‐binding domain of HsHAD or (b) superimposing the D/E domains of rpMFE1 on the two assembled dimerization domains of the HsHAD dimer.

## Results and discussions

### The rpMFE1 enzyme kinetic studies with the short‐chain 2E‐butenoyl‐CoA and AcAc‐CoA substrates

The enzyme kinetic data for short‐chain substrates (having a four‐carbon atom acyl chain) are summarized in Table 2. These kinetic data have been measured with 2E‐butenoyl‐CoA (also known as crotonyl‐CoA, in the forward direction) and AcAc‐CoA (in the reverse direction) as the substrates. The forward hydratase and the forward NAD^+^ dependent dehydrogenase reaction rates are at least 10‐fold lower than the rates for the corresponding monofunctional enzymes 35, 36, 37. The *k*_cat_ for the hydratase reaction (113 s^−1^) is much higher than the *k*_cat_ for the forward dehydrogenase reaction (1.7 s^−1^), as previously observed also for substrates with longer acyl chains (Table 2), whereas the *K*_m_ values for the hydratase and forward dehydrogenase reactions are 82 and 3.6 μm, respectively. The *k*_cat_ and *K*_m_ values for the reverse dehydrogenase reaction, using AcAc‐CoA as substrate, are, respectively, 23 s^−1^ and 44 μm, having therefore a *k*_cat_ that is also about 10‐fold lower than of the monofunctional enzyme. In the forward dehydrogenase assay of MFE1, the substrate is generated by its hydratase active site, which therefore functions as a linker enzyme, generating the dehydrogenase substrate. As the hydratase *k*_cat_ is much higher than the forward dehydrogenase *k*_cat_ for the short‐chain substrate, it can be assumed that in this assay (Table 2) the hydratase reaction is not the rate‐limiting step, being independent from possible substrate channeling. Substrate channeling of MFE1 has been reported in case the substrate is 2E,4E‐decadienoyl‐CoA, for which the *k*_cat_ and *K*_m_ values of the hydratase reaction were determined to be 1.7 s^−1^ and 24 μm, respectively 16 (Table 2), whereas for the 2E‐decenoyl‐CoA substrate the *k*_cat_ and *K*_m_ values of the hydratase reaction were determined to be 80 s^−1^ and 21 μm, respectively 38 (Table 2).

**Table 2:** The Michaelis–Menten kinetic constants of the hydratase and the dehydrogenase activities of MFE1 and of the corresponding monofunctional enzymes

Enzyme, assay	Substrate	*k*_cat_ (s^−1^)	*K*_m_ (μm)	*k*_cat_/*K*_m_ (m ^−1^·s^−1^)	Comments	References
rpMFE1 (short‐chain substrates)						
Hydratase	2E‐Butenoyl‐CoA	113.0 ± 33.0	82.0 ± 18.0	1.4*10^6^	pH 9.0	This work
Dehydrogenase, forward	3S‐Hydroxybutanoyl‐CoA	1.7 ± 0.1	3.6 ± 1.0	0.5*10^6^	pH 9.0	This work
Dehydrogenase, reverse	AcAc‐CoA	23.0 ± 2.3	44.0 ± 15.0	0.5*10^6^	pH 7.0	This work
rpMFE1 (medium‐chain substrates)						
Hydratase	2E‐Hexenoyl‐CoA	102	9	11*10^6^	pH 8.0, linked assay: HAD	38
Hydratase	2E‐Decenoyl‐CoA	80	21	3.8*10^6^	pH 8.0, linked assay: HAD	38
Hydratase	2E,4E‐Decadienoyl‐CoA	1.7	24	0.07*10^6^	pH 8.0, linked assay: HAD and thiolase	16
Dehydrogenase, forward	3S‐Hydroxyhexanoyl‐CoA	22	11	2.0*10^6^	pH 10.2, linked assay: hydratase	7
Monofunctional enzymes (short‐chain substrates)						
Bovine hydratase	2E‐Butenoyl‐CoA	1000	40	25.0*10^6^	pH 7.5	37, 46
Bovine HAD, forward	3S‐Hydroxybutanoyl‐CoA	200	75	2.7*10^6^	pH 10.0	36
HsHAD, reverse	AcAc‐CoA	250	15	16.7*10^6^	pH 7.0	35

### The structure of the hydratase active site of rpMFE1 complexed with AcAc‐CoA

**Figure 3:**
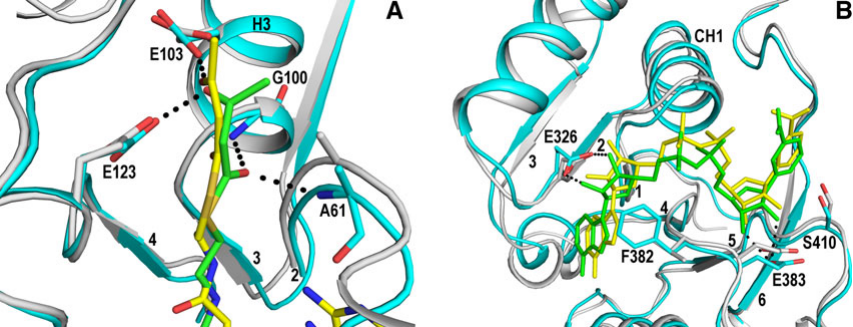
Comparison of the structures of the liganded active sites of MFE1 and its monofunctional counterparts. (A) The hydratase active site. Comparison of the mode of binding of AcAc‐CoA to the hydratase active site of MFE1 (cyan, 5MGB) and to the active site of the monofunctional hydratase (gray, 1DUB). The MFE1 residues are labeled. The oxyanion hole of MFE1 is formed by N(Ala61) (loop‐2) and N(Gly100) (helix‐3). The catalytic residues are Glu103 and Glu123. H3 identifies the active site helix (helix‐3) and the numbers 2, 3, and 4 identify loop‐2 and β‐strands B3 and B4 of domain A, respectively. Dotted lines (black) mark the hydrogen bonds of the thioester and the 3‐keto oxygen atoms with the oxyanion hole peptide NH‐groups and the catalytic glutamates, respectively. (B) The mode of binding of NAD ^+^ in the structures of binary complexes. For this comparison, domain C of MFE1 (cyan, 5MGB) has been superimposed on the NAD‐binding domain of HsHAD (gray, 3HAD). CH1 identifies the helix that interacts with the NAD pyrophosphate moiety and the numbers 1 to 6 identify the β‐strands CB1 to CB6 of the Rossmann fold of domain C. Glu326 interacts with the ribose moiety of the ADP part of NAD ^+^. The side chain of Phe382 contacts also the ADP‐ribose moiety. Glu383 is hydrogen bonded to the nicotinamide‐ribose moiety and to the main chain of the loop after CB5, identified by the labeled Ser410 (which corresponds to Ser137 of HsHAD). Dotted lines (black) mark the hydrogen bond interactions of the Glu326 and Glu383 side chains, respectively, with the ADP‐ribose and the nicotinamide‐ribose moieties of NAD ^+^.

In the AcAc‐CoA/NAD^+^ complex structure, refined at 2.8 Å resolution (Table 1), the AcAc‐CoA is only bound in the hydratase active site. Its mode of binding is well defined by the electron density map (Fig. S2), adopting its characteristic bent conformation. The thioester oxygen of AcAc‐CoA is hydrogen bonded to the peptide NH‐groups of Ala61 (loop‐2, Fig. S1) and Gly100 (N terminus of the helix‐3) (Fig. 3A). The 3‐keto oxygen is hydrogen bonded to the carboxylate oxygens of Glu103 and Glu123, like in the complex of the monofunctional hydratase 32, suggesting that the carboxylate moieties of both catalytic glutamates are protonated in this complex. The acetoacetyl moiety is bound in a somewhat twisted conformation (Fig. 3A), whereas it is bound in a planar conformation in the active site of the monofunctional hydratase 32. The twisted conformation of the acetoacetate moiety is not possible in the monofunctional hydratase active site, due to the presence of the more bulky side chain of Trp120 in this active site, which is Leu75 (helix‐2) in MFE1 (Fig. S3). In molecule B, the side chain of Leu73 (of the flexible loop‐2) is also pointing into the active site, but in molecule A, due to conformational differences in loop‐2, correlated with different crystal packing, the Leu73 side chain is pointing outward. In the crotonase fold enzymes, the active site is covered by the C‐terminal helix, helix‐10, which in MFE1 functions also as the linker helix, domain B (Fig. 2). Sequence conservation suggests that a basic residue of helix‐10 is important for its function, forming a salt bridge with a phosphate group of the substrate. Also, a hydrophobic residue pointing to the pantetheine moiety of CoA is conserved 39, 40 in the crotonase fold. In the MFE1 helix‐10, the basic residue, Lys275, forms a salt bridge with the pyrophosphate group of the CoA moiety and the hydrophobic residue, pointing to the pantetheine moiety, is Phe271. Phe271 is part of a conserved hydrophobic cluster that includes Ile63 and Leu126 (Fig. S3). Ile63 and Leu126 protrude out of loop‐2 and loop‐4, respectively, anchoring helix‐10 to domain A of the crotonase fold.

### The structure of the HAD active site of rpMFE1 complexed with NAD^+^

**Figure 4:**
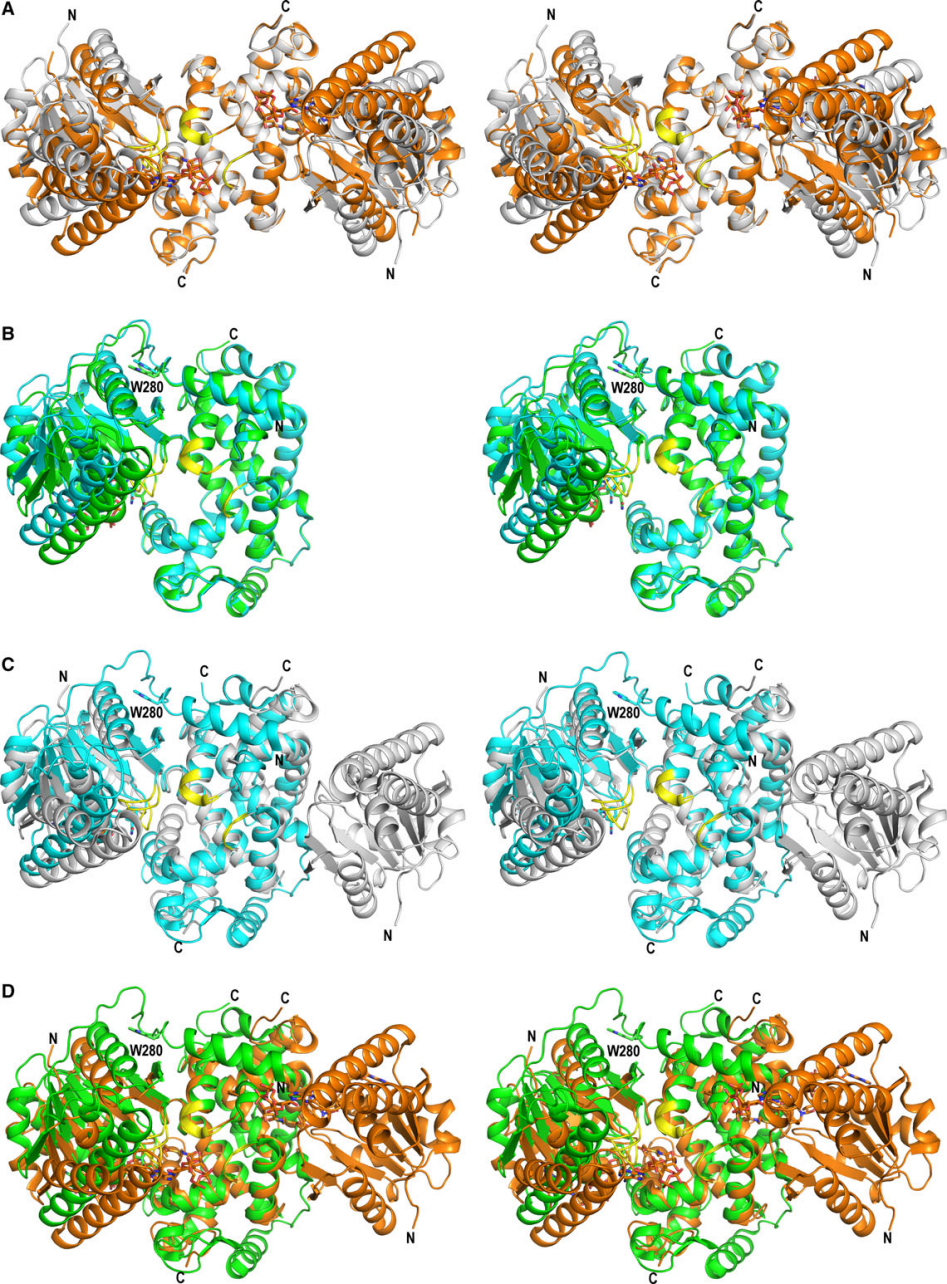
The open and closed conformations of domain C with respect to the D/E domains. Domain C of MFE1 corresponds to the NAD‐binding domain of HsHAD. The D/E domains of MFE1 correspond to the two assembled dimerization domains of HsHAD. The latter MFE1 and HsHAD units have been superimposed on each other in each of the panels. The view is toward the active site along the dimer twofold axis of HsHAD. The visualized MFE1 Cα‐trace includes the B, C, D, and E domains. W280 identifies Trp280 at the C‐terminal end of domain B. The HsHAD Cα‐trace covers the complete dimer. Residues of the Cβ6‐Cβ7 loop (of the NAD‐binding domain in HsHAD, domain C in MFE1), and of the two DH2‐DH3 loops (of the dimerization domains in HsHAD, domains D/E in MFE1) shape the pantetheine binding tunnel (P‐tunnel) of HsHAD and are highlighted in yellow. (A) Comparison of the unliganded, open structure of HsHAD (gray, 1F14) and liganded, closed structure of HsHAD (orange, 1F0Y, including the AcAc‐CoA ligand and NAD ^+^). (B) Comparison of molecule B of MFE1 (cyan, 5MGB, fully open) and molecule A of MFE1 (green, 5MGB, partially closed). (C) Comparison of molecule B of MFE1 (cyan, 5MGB, fully open) and the unliganded, open conformation of HsHAD (gray, 1F14). (D) Comparison of molecule A of MFE1 (green, 5MGB, partially closed) with the liganded fully closed conformation of HsHAD (orange, 1F0Y, including the AcAc‐CoA ligand and NAD ^+^).

NAD^+^ is bound to the C domain, in a deep cleft between the C domain and the D/E domains (Fig. 2, Fig. S4). The mode of binding of NAD^+^ to the HAD active site is well defined by the respective electron density map (Fig. S2), except for the nicotinamide moiety whose atoms have relatively high B factors, like in HsHAD 22. The NAD^+^ mode of binding to MFE1 is the same as for NADH, which is also well defined by its electron density, including its nicotinamide moiety (Fig. S2) 25 and which interacts with the side chain of the conserved Met307 (Fig. S5). The affinity of MFE1 for NADH (*K*_d _= 1 μm) is much higher than for NAD^+^ (*K*_d _= 120 μm) 7, like also reported for HsHAD 34. The mode of binding of NAD^+^ to MFE1 is also very similar as seen in HsHAD (Fig. 3B, Fig. S5). The dimeric HsHAD, consisting of two identical subunits, has a broad substrate specificity, such that the length of the acyl chain of its substrate, 3S‐hydroxyacyl‐CoA, extends from 4 to 16 carbons 36. Each subunit adopts a bilobal fold. The sequence alignment of HsHAD and rpMFE1 is given in Fig. S1. The sequence identity between the HAD part of rpMFE1 and HsHAD is 31%. Compared to MFE1, HAD is missing the A, B, and E domains of MFE1 (Fig. S1). The N‐terminal, NAD‐binding domain of HsHAD corresponds to the MFE1 C domain and the two C‐terminal dimerization domains of the HsHAD dimer correspond to the MFE1 D and E domains, respectively (Fig. 4). Structural studies of HsHAD have provided the crystal structures of HsHAD complexed with NAD^+^, NADH, substrate (3S‐hydroxybutanoyl‐CoA), and a dead‐end ternary complex with bound NAD^+^ and AcAc‐CoA 22. In the latter complex, the N‐terminal domain has moved toward the dimerization domain, such that HsHAD has adopted its fully closed conformation (Fig. 4), required for being able to catalyze its reaction. In this ternary complex, the pantetheine part of AcAc‐CoA is bound in an extended tunnel (P‐tunnel) between the CB6‐CB7 loop of the NAD‐binding domain, and the two DH2‐DH3 loops of the HsHAD dimerization domains (Fig. 4).

In the AcAc‐CoA/NAD^+^ complex structure of MFE1, NAD^+^ only interacts with the C domain (Fig. S5) and no domain closure is induced by its binding. The pyrophosphate moiety binds at the N terminus of helix CH1 of the N‐terminal βαβ‐unit (CB1‐CH1‐CB2, Fig. 3B) of the Rossmann fold of the C domain. As is observed in other NAD‐binding βαβ‐units 41, 42, a glutamate at the end of the second β‐strand (CB2, Glu326) interacts with the ribose moiety of the adenosine part. The side chain of Phe382, at the end of the CΒ4 β‐strand, swings from a solvent‐exposed conformation in the absence of bound NAD toward covering the ADP‐ribose moiety in the presence of NAD^+^ as well as NADH (Fig. 3B, Fig. S4). No other conformational changes are induced on binding NAD^+^ or NADH. A second glutamate (Glu383, also at the end of CB4) interacts with the hydroxyl groups of the nicotinamide‐ribose group. The Glu383 side chain (corresponding to Glu110 in HsHAD) is also anchored to N(Ser410) and N(Ala411) of the loop after CB5 (Fig. S5). These hydrogen bond interactions have also been described for the structures of the HsHAD binary complexes. Ser410 corresponds to Ser137 in HsHAD, and in HsHAD, this ‘Ser137‐loop’ adopts a different conformation in its ternary complex 22.

### The conformational flexibility of MFE1

**Figure 5:**
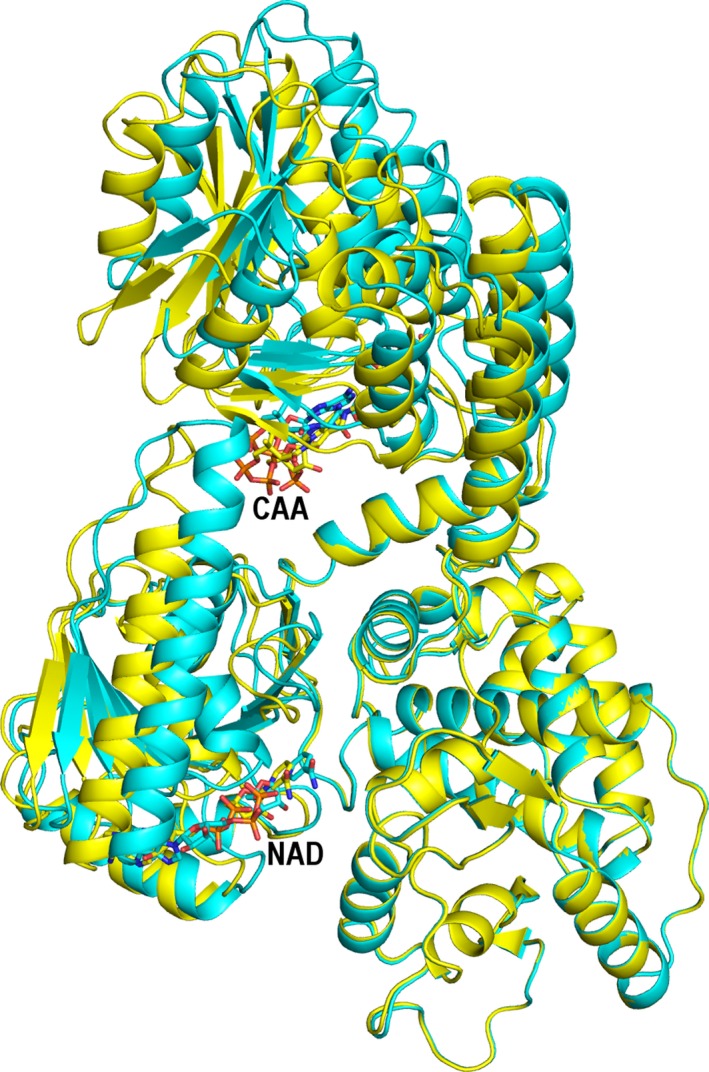
The conformational flexibility of MFE1. Comparison of molecule A (cyan) and molecule B (yellow), such that the D/E domains of both molecules (5MGB) have been superimposed. The bound AcAc‐CoA (labeled CAA, domain A, upper part) and NAD ^+^ (labeled NAD, bound to domain C, in the groove between domain C and the D/E domains, lower part) are presented as sticks using atom‐type coloring.

In the crystal form of MFE1 used here for its structural characterization, the conformations of the HAD part of molecules A and B are different (Fig. 5). These conformational differences are related to differences in crystal packing and these conformational differences are seen in each of the available rpMFE1 crystal structures (Table 3). In molecule A, the HAD part adopts a partially closed conformation, whereas in molecule B it is fully open, because of a rotation of domain C, away from the D/E domains (Video S1). This is a rigid body rotation. Superpositioning of domain C of molecules A and B results in an rmsd for corresponding Cα atoms of 0.2 Å (excluding only the tip of the flexible CH2‐helix). The conformational differences in the HAD part can be quantified by measuring the distance between Cα(Thr306) (N‐terminal CH1 helix) and Cα(Ala524) (C‐terminal DH3 helix) (Fig. S4, Table 3). These two residues are located on the opposite sides of the cleft that binds the NAD cofactor. Helices CH1 and DH3 point away from the cleft (Fig. S4). The distances between the residues at the C terminus and N terminus of helices CH1 and DH3, respectively, of molecules A and B are also listed (Table 3). The residues in HsHAD corresponding to Thr306 and Ala524 are Leu25 and Val253, respectively (Fig. S1). The distance between these Cα atoms is 8.3 Å in the fully closed HsHAD active site and 12.0 Å for the fully open HsHAD active site. For the MFE1 active site, this distance is 10.2 Å for the molecule A of the AcAc‐CoA/NAD^+^ complex and it is 12.8 Å for its molecule B (Table 3). In the HsHAD ternary complex, the NAD‐binding domain has moved further toward the dimerization domain than in molecule A of the MFE1 complex, adopting a fully closed conformation with tight stacking interactions between the nicotinamide group and the 3‐keto group 22. The hinge region of this motion appears to be the regions 201–207 in HsHAD 35 and 474–480 in rpMFE1 (Fig. 4).

**Table 3:** Conformational differences between the active sites of molecule A and molecule B^a^

Crystal form	The AcAc‐CoA/NAD^+^ complex crystal form, with bound AcAc‐CoA and NAD^+^ (this paper)	The 3S‐hydroxydecanoyl‐CoA complex crystal form, with bound 3S‐hydroxydecanoyl‐CoA and NADH 25	The unliganded crystal form 25	The CoA complex crystal form with bound CoA 20
Experimental conditions	Cocrystallization experiment with AcAc‐CoA and NAD^+^	Crystal first grown as the CoA complex. Subsequently obtained after the washing steps, followed by a soaking experiment with 2E‐decenoyl‐CoA and NADH	Crystal first grown as the CoA complex. Subsequently obtained after washing steps, by which CoA is removed	Crystal grown by cocrystallization in the presence of CoA
PDB entry code	5MGB	3ZWC	3ZW8	2X58
Hydratase active site (molecule A)				
Active site ligand	AcAc‐CoA	3S‐Hydroxydecanoyl‐CoA	Sulfate	CoA
Gly100‐Phe271^b^	17.3	17.6	17.5	17.9
Cys108‐Trp280^b^	42.0	42.4	42.2	42.8
Dehydrogenase active site (molecule A)				
Active site ligand	NAD^+^	NADH	Water	ADP
Thr306‐Ala524^c^	10.2	10.2	10.9	11.1
Ala316‐Phe518^c^	33.1	33.0	33.1	33.3
Hydratase active site (molecule B)				
Active site ligand	AcAc‐CoA	3S‐Hydroxydecanoyl‐CoA	Sulfate	CoA
Gly100‐Phe271^b^	16.6	16.9	17.1	16.9
Cys108‐Trp280^b^	40.4	40.7	40.7	40.7
Dehydrogenase active site (molecule B)				
Active site ligand	NAD^+^	NADH	Sulfate	Sulfate
Thr306‐Ala524^c^	12.8	12.9	13.6	13.5
Ala316‐Phe518^c^	34.9	34.9	34.9	34.9

a The crystal packing in each of these structures is the same.

b The listed distance (in Å) is between the Cα atoms of the given residues. These residues are also identified in Fig. 2.

c The listed distance (in Å) is between the Cα atoms of the given residues. These residues are also identified in Fig. S4.

It can be noted that for each of the four structures, the HAD active site of molecule A is more closed than in molecule B, whereas its hydratase active site is more open.

This rigid body motion toward a more closed conformation of domain C is correlated with an upward movement of domain A, adopting a more open conformation, away from the HAD part (Fig. 5, Video S1) in molecule A. The domain A movement is also a rigid body motion. Superpositioning of domain A of molecules A and B results in an rmsd for corresponding Cα atoms of 0.3 Å (excluding only the flexible loop‐2 region). The conformational difference is also seen from the distance information provided in Table 3. The distance between Cα(Gly100) and Cα(Phe271) is 17.3 Å in molecule A, and it is 0.7 Å shorter in molecule B. This distance measures the distance between the N terminus of the active site helix‐3 and the middle part of the linker helix, Phe271, whose side chain contacts the bound substrate (Fig. 2). The hinge region is near the N terminus of domain B (Video S1, Fig. 5). In this conformational movement, domain A of molecule A moves away from the linker helix and a comparison of distances between atoms further away from the hinge region (between Cα(Cys108), at the end of helix‐3, and Cα(Trp280), at the end of the linker helix) show larger differences, from 42.0 Å in molecule A to 40.4 Å in molecule B.

Clearly, MFE1 has two hinge motions, being the movement of the A domain with respect to the BCDE part (more open in molecule A) and the open/closed conformational switch of the HAD part (more closed in molecule A), being the movement of the C domain with respect to the D/E domains. The structure comparisons with HsHAD (Fig. 4) suggest that the HAD part of molecule A is only partially closed in this crystal form. In this partially closed form, domain A moves away from domain B (Table 3). Domain B is an integral part of the crotonase fold, being helix‐10. Experimental studies have addressed the importance of domain B (helix‐10) for full MFE1 activity. The interactions of the B domain (helix‐10) with the D/E domains are important for full hydratase activity of MFE1, which was shown by extensive MFE1 deletion experiments, as it was found that a deletion mutant in which the C domain was deleted still has full hydratase activity 38, whereas constructs without the D/E part have lost the hydratase activity. Additionally, point mutation studies of helix‐10 of a model enzyme of the crotonase superfamily fold have shown the critical importance of helix‐10 for the catalytic properties of this model enzyme. In this model enzyme, Δ^3^,Δ^2^‐enoyl‐CoA isomerase, the residue that corresponds to Phe271 (Fig. 2) in MFE1, was mutated into an alanine, and this point mutation variant was much less active than wild‐type 39, 40. Further rotation of the C domain of MFE1 toward a fully closed HAD structure will likely move the A domain further from its HAD BCDE part and from its helix‐10 (domain B), and therefore this will affect the interactions between domain A and its helix‐10, being an integral part of the HAD part of MFE1. These structural changes are therefore expected to affect the binding and catalytic properties of the hydratase active site of MFE1. Therefore, the correlated motions of the HAD part and the A domain could function as an allosteric control mechanism 43, 44 between the dehydrogenase and hydratase active sites, facilitating substrate channeling of tightly bound intermediates by inducing the release of the product of the hydratase active site when the dehydrogenase active site is complexed with NAD^+^. In this respect, it is interesting that for HsHAD the binding of NAD^+^ precedes the binding of substrate and the affinity for its substrate is much increased after binding of NAD^+^ 22.

## Concluding remarks

In MFE1, the hydratase and dehydrogenase active sites are built by one polypeptide chain. The conformational flexibility of these two parts with respect to each other provides additional complexity to the catalytic properties of MFE1, as compared to the corresponding monofunctional homologues. This additional complexity correlates with lower catalytic rates of MFE1 for short‐chain substrates, being 10‐fold (or more) lower than of these counterparts. The presence of open and closed conformations of the HAD part of MFE1 is in common with the monofunctional HAD. However, in this MFE1 crystal structure, obtained by cocrystallization in the presence of AcAc‐CoA and NAD^+^, the fully closed, ternary complex conformation, as seen in HsHAD, is not captured. AcAc‐CoA is only bound in the hydratase active site and not in the HAD active site. Further structural studies of rpMFE1 aimed at capturing the fully closed conformation of its HAD part have been initiated.

## Author contributions

PK and RKW planned the experiments; PK purified the protein and performed the crystallography experiments; PK, GBM, and SS performed the enzyme kinetic experiments; WS prepared reagents; PK, TRK, and RKW analyzed the structures and prepared the figures. RW, PK, TRK, and JKH wrote the manuscript.

## Supporting information


**Fig. S1.** The sequence alignment of rat peroxisomal MFE1 (rpMFE1) and human mitochondrial HAD (HsHAD).
**Fig. S2.** The 2Fo‐Fc electron density omit maps defining the mode of binding of the active site ligands.
**Fig. S3.** Comparison of the mode of binding of AcAc‐CoA to the hydratase active sites of MFE1 and the monofunctional hydratase.
**Fig. S4.** The structure of the HAD part of MFE1 in complex with NAD^+^.
**Fig. S5.** Comparison of the mode of binding of NAD^+^ to the NAD‐binding domains of the binary complexes of MFE1 and HsHAD.

**Video S1.** The conformational flexibility of MFE1.
