# Supplementary material for: Structural enzymology comparisons of multifunctional enzyme, type‐1 (MFE1): the flexibility of its dehydrogenase part
Source: FEBS Open Bio. 2017 Nov 6;7(12):1830–42. doi: 10.1002/2211-5463.12337 (PMC5715344; doi:10.1002/2211-5463.12337)
Supplement: Supplementary file 1 — Fig. S1. The sequence alignment of rat peroxisomal MFE1 (rpMFE1) and human mitochondrial HAD (HsHAD). Fig. S2. The 2Fo‐Fc electron density omit maps defining the mode of binding of the active site ligands. Fig. S3. Comparison of the mode of binding of AcAc‐CoA to the hydratase active sites of MFE1 and the monofunctional hydratase. Fig. S4. The structure of the HAD part of MFE1 in complex with NAD+. Fig. S5. Comparison of the mode of binding of NAD+ to the NAD‐binding domains of the binary complexes of MFE1 and HsHAD. [file FEB4-7-1830-s001.pdf]

Structural enzymology comparisons of multifunctional enzyme, type-1 (MFE1): the flexibility of its dehydrogenase part

Prasad Kasaragod[1], Getnet B. Midekessa[1], Shruthi Sridhar[1], Werner Schmitz[2], Tiila-Riikka Kiema[1], J. Kalervo Hiltunen[1] and Rik K. Wierenga[1]

[1]Biocenter Oulu and Faculty of Biochemistry and Molecular Medicine, P.O. Box 5400, FI-90014, University of Oulu, Finland
[2]Theodor Boveri Institute of Biosciences (Biocenter), University of Würzburg, Am Hubland, D-97074, Würzburg, Germany
Corresponding author: Rik K. Wierenga, E-mail: rik.wierenga@oulu.fi

Supplementary material

Movie S1. The conformational flexibility of MFE1.

Fig. S1. The sequence alignment of rat perixosomal MFE1 (rpMFE1) and human mitochondrial HAD (HsHAD).

Fig. S2. The 2Fo-Fc electron density omit maps defining the mode of binding of the active site ligands.

Fig. S3. Comparison of the mode of binding of AcAc-CoA to the hydratase active sites of MFE1 and the monofunctional hydratase.

Fig. S4. The structure of the HAD part of MFE1 in complex with NAD^+.

Fig. S5. Comparison of the mode of binding of NAD^+ to the NAD-binding domains of the binary complexes of MFE1 and HsHAD.

Movie S1. The conformational flexibility of MFE1. Comparison of molecule A (cyan) (5MGB) and molecule B (yellow) (5MGB), such that the D/E-domains of both molecules have been superimposed on each other. The bound AcAc-CoA (domain A, upper part) and NAD^+ (bound to domain C in the groove between domain C and the D/E-domains, lower part) are included, and shown as sticks using atom-type coloring.

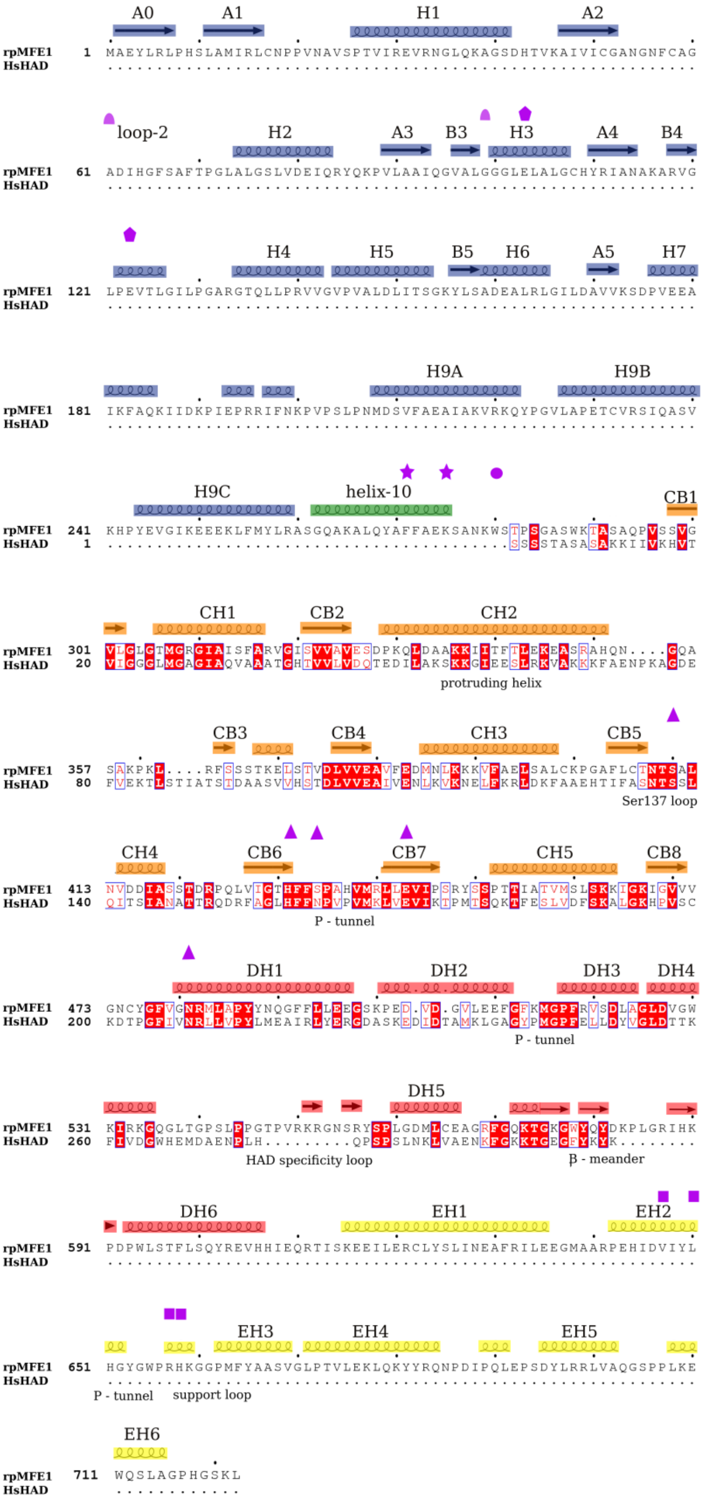

Fig. S1. The sequence alignment of rat peroxisomal MFE1 (rpMFE1) and human mitochondrial HAD (HsHAD). The secondary structure of rpMFE1 is shown above the sequences. ◖ identifies the two oxyanion hole residues of the hydratase active site, ⬟ identifies the two catalytic glutamates of the hydratase active site, ★ labels the helix-10 residues that point to the CoA moiety of the ligand bound in the active site of the hydratase domain, ● highlights Trp280 at the C-terminal end of helix-10, ▲ labels Ser410, His431, Ser434, Glu443 and Asn481 which are the catalytic residues of the dehydrogenase domain. ■ marks the residues of the E-domain that interact with the N-terminus of helix-10. CB1, CB2, CB3, CB4, CB5 and CB6 form the six β-strands of the Rossmann-fold of the NAD-binding domain C, being extended by β-strands CB7 and CB8.

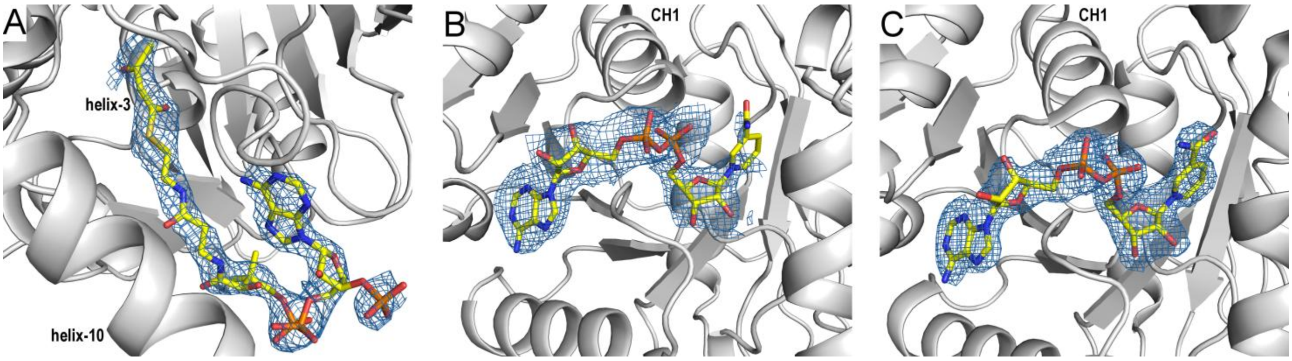

Fig. S2. The 2Fo-Fc electron density omit maps defining the mode of binding of the active site ligands, calculated after 10 cycles of omit refinement. A) AcAc-CoA (5MGB, contour level 1.0 sigma). B) NAD^+ (5MGB, contour level 0.7 sigma). C) NADH (3ZWC, contour level 1.0 sigma).

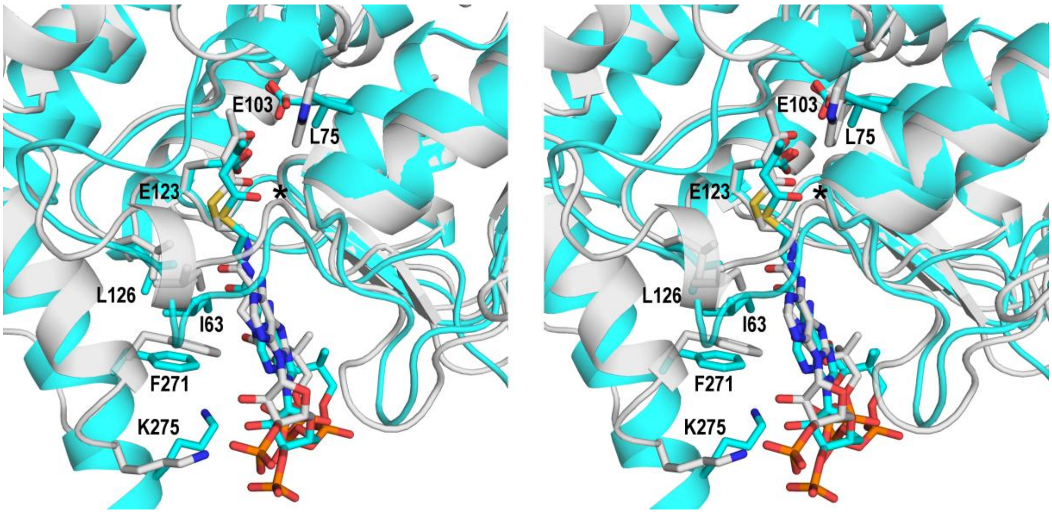

Fig. S3. Comparison of the mode of binding of AcAc-CoA to the hydratase active site of MFE1 (cyan, 5MGB) and to the active site of the monofunctional hydratase (grey, 1DUB). The MFE1 residues are labeled. The * labels the oxyanion hole of MFE1, formed by N(Ala61) (loop-2), and N(Gly100) (helix-3). The catalytic residues are Glu103 and Glu123. Phe271 and Lys275 protrude out of helix-10 and point towards the substrate binding site. Phe271, together with Ile63 (loop-2) and Leu126 (loop-4), is part of a hydrophobic cluster near the thioester moiety of AcAc-CoA. Leu75, corresponding to Trp120 of the monofunctional hydratase, points towards the acyl tail of AcAc-CoA.

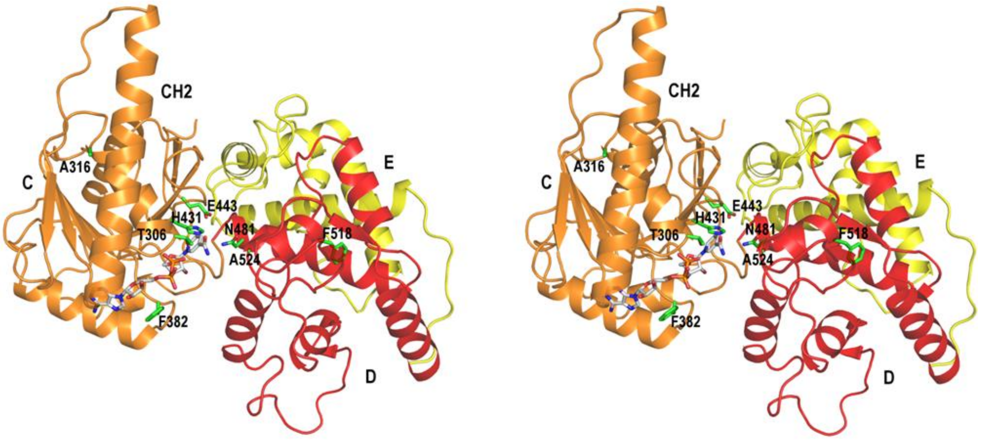

Fig. S4. The structure of the HAD part of MFE1 (5MGB), complexed with NAD^+, using the same color code as in Fig. 2. The labeled residues Thr306, Ala316, and Phe518, Ala524 identify the beginning and end of the helices CH1 and DH3, respectively. The residues His431, Glu443 and Asn481 identify the dehydrogenase active site. The side chain of Phe382 contacts the ADP-ribose moiety.

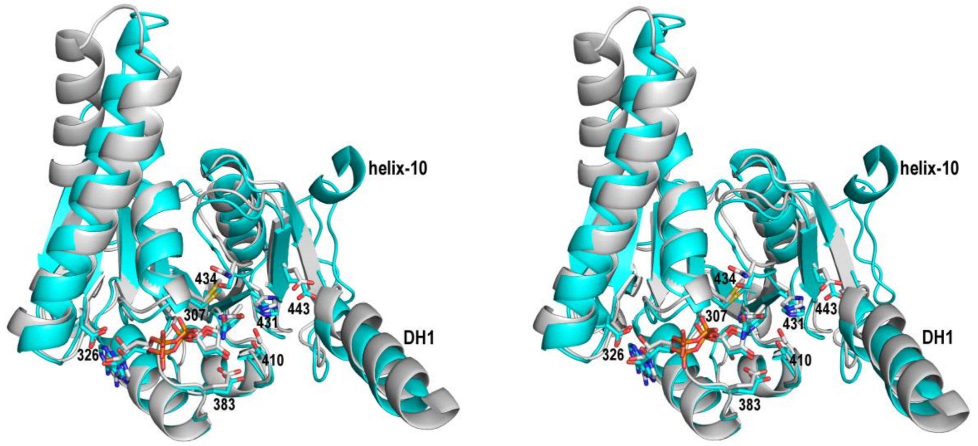

Fig. S5. Comparison of the mode of binding of NAD^+ to the NAD-binding domains of the binary complexes of MFE1 (cyan, 5MGB) and HsHAD (grey, 3HAD), after superpositioning of these domains on each other. The residues of the catalytic dyad His431 (CB6) and Glu443 (CB7) are labeled. Also labeled are Ser410 of the "Ser137-loop", after CB5. This loop is anchored to Glu383 (in the loop after CB4). Met307 is at the N-terminus of helix CH1. Glu326, after CB2, is hydrogen bonded to the ADP-ribose moiety of NAD^+. Glu383 is also hydrogen bonded to the nicotinamide-ribose group. Helix-10 labels the linker helix and DH1 identifies the first helix of domain D.
